# Digital health diplomacy and universal health coverage

**DOI:** 10.2471/BLT.24.291846

**Published:** 2024-12-11

**Authors:** Jarbas Barbosa da Silva, Mary Lou Valdez, Rhonda Sealey-Thomas, Socorro Gross Galiano, Sebastian Garcia-Saisó, Piedad Huerta, Luis Jimenez McInnis, Mariana Faria Teixeira, Myrna Marti, Ana Estela Haddad, Marcelo D’Agostino

**Affiliations:** a Office of the Director, Pan American Health Organization, 525 23rd Street NW, Washington, DC 20037, United States of America (USA).; b Pan American Health Organization Office, Brasilia, Brazil.; c Department of Evidence and Intelligence for Action in Health, Pan American Health Organization, Washington, DC, USA.; d Department of Country and Sub-regional Coordination, Pan American Health Organization, Washington, DC, USA.; e Department of External Relations, Pan American Health Organization, Washington, DC, USA.; f Information and Digital Health, Ministry of Health, Brazil.

In July 2018, the United Nations (UN) Secretary-General convened a high-level panel on digital cooperation to propose strategies for optimizing the use of digital technologies, and mitigating associated risks through collaboration among governments, the private sector, civil society and international organizations. The Panel’s key recommendations included building an inclusive digital economy, developing capacity, protecting human rights, promoting digital security and fostering global cooperation.^1^ The coronavirus disease 2019 (COVID-19) pandemic accelerated the shift to virtual platforms, which created opportunities for change in diplomacy, policy-making, health care and societal engagement.^2^ However, considerable disparities in access to digital health solutions and lack of connectivity and inclusive digital health policies persist, particularly in low- and middle-income countries, making it a diplomatic challenge that requires global action.

Digital diplomacy, defined as the use of digital tools by governments to enhance international cooperation, contributes to addressing these challenges. Digital health diplomacy, a subset of diplomacy, uses these tools to foster international collaboration and ensure equitable access to digital health innovations, particularly in underserved regions.

While frameworks like the UN’s *Roadmap for digital cooperation*^1^ and the World Health Organization’s (WHO) *Global strategy on digital health 2020–2025 *^3^ highlight the importance of digital health, the role of diplomacy in promoting equitable access to digital health and solutions remains underexplored. This article seeks to fill that gap by emphasizing digital health diplomacy as an important mechanism for bridging the global digital divide and promoting health equity under the premise that without structured multilateral engagement, even advanced digital health innovations may fail to reduce disparities.

Digital diplomacy and international collaboration offer a path to globally equitable health systems, as shown in the 2024 Group of 20 (G20), whose focus on digital health aligns international health policies with ongoing transformation efforts. The COVID-19 pandemic demonstrated the importance of collaboration among governments, with countries sharing real-time data on vaccine distribution and efficacy. A multistakeholder approach to digital health diplomacy can therefore create a healthier, more resilient world.

The health sector must collaborate with foreign affairs departments and multilateral organizations to prioritize the needs of the most vulnerable, and use digital transformation to promote social justice and health equity.^4^ In this article, we advocate for a renewed focus on digital health diplomacy, highlighting its transformative role in achieving these outcomes, and urging governments and stakeholders to work together to ensure that digital health innovations contribute to resilient, accessible and equitable health systems.

## Digital transformation 

The digital transformation of the health sector provides opportunities to address global health challenges by improving service delivery, access and efficiency. Innovations such as electronic health records, artificial intelligence, telehealth and updated regulations are making health care more accessible and equitable.^5^ For example, a technical and diplomatic collaboration between Brazil’s health ministry, the Pan American Health Organization (PAHO) and international partners extended telehealth services to remote Amazonian communities, improving access and addressing disparities. Telemedicine and mobile health applications (apps) help overcome barriers like financial constraints, geographical isolation, stigma and discrimination, bridging gaps in care and enhancing disease surveillance. Prioritizing digital transformation is essential for building equitable and inclusive health-care systems.

Diplomacy must now address emerging technologies such as artificial intelligence, cybersecurity and digital legal frameworks at bilateral, multilateral and global levels. As digital governance becomes integral to negotiations on health, trade and human rights, diplomats must be trained to navigate these evolving digital landscapes.^6^ The rise of digital diplomacy reflects the shift towards international collaboration and digital transformation to address global health disparities and promote equity. Governments use digital networks to support health initiatives, share data and conduct negotiations across borders, emphasizing multistakeholder engagement. During the COVID-19 pandemic, digital diplomacy advanced international cooperation through initiatives like COVAX where governments and partners used digital platforms to coordinate vaccine distribution. These tools enabled virtual meetings, real-time data sharing and delivery monitoring, improving transparency and efficiency in reaching underserved populations. While digital diplomacy enhances accessibility, speeds decision-making and reduces costs,^7^ it also faces challenges such as the digital divide, cybersecurity risks, loss of personal interaction and technical limitations. Despite these issues, digital diplomacy remains a valuable complement to traditional diplomacy, driving collaboration and innovation in global problem-solving.

Digital platforms enhance transparency, foster public trust and enable inclusive participation, while tools like social media, virtual reality and artificial intelligence improve communication, crisis management and decision-making. As a promotor of cultural exchange and mutual understanding, digital diplomacy can leverage big data analytics to get insights into public opinion and policy guidance and use technological advancements for a common good.^8^^,^^9^

The synergy between digital transformation and digital diplomacy provides an important opportunity to improve health outcomes while promoting international peace, security and cooperation. Governments’ commitment to digital diplomacy, combined with the co-creation of digital health public goods such as digital vaccine certificates or telehealth platforms, would enhance the impact of digital health innovations, contributing to more resilient, equitable and sustainable health systems. These two areas working together can create a pathway towards a global health ecosystem that is technologically advanced and universally accessible. Advocacy for digital health solutions, led by diplomats and foreign affairs institutions and networks, and supported by governments’ dedication to digital diplomacy, bridges the digital divide and contributes to global access to quality health care. The role of digital diplomacy and multistakeholder collaboration in global health is critical because it proposes that governments, private sector, civil society, academia and international organizations work together to harness the full potential of digital transformation. This collaboration is important for developing strong governance frameworks, enhancing digital literacy and ensuring that technological advancements benefit all segments of the population.

Recognizing the impact of digital transformation, PAHO integrated digital health innovations into diplomatic efforts through launching the *Sustainable Health Agenda for the Americas 2018–2030*,^10^ which emphasizes enhancing information systems and digital health. In 2019, PAHO and its Member States endorsed a Pan-American call to action, establishing eight guiding principles^11^ and a regional roadmap for digital transformation.^12^ Inspired by the UN’s digital cooperation framework, PAHO was among the earliest organizations to align diplomatic, political and technical strategies for digital health across borders. In 2021, the WHO* Global Strategy on Digital Health 2020–2025* underscored digital diplomacy as a cross-border approach to building equitable and sustainable digital health ecosystems.^3^ Digital health became a Group of 20 (G20) priority in 2023, with WHO launching the global initiative on digital health during India’s G20 Presidency in 2023. Brazil’s G20 Presidency in 2024 prioritized telehealth and health data integration and highlighted the potential of digital technologies in health care. These efforts align with sustainable development goal 17, which aims to strengthen global partnerships. Digital health diplomacy, through collaboration among governments, private sectors and international organizations, is vital for fostering these partnerships and ensuring that digital transformation benefits all nations equitably.

Digital transformation in health faces considerable challenges, particularly in low-income countries that lack infrastructure such as electricity, internet access and digital literacy. Without these foundations, digital health tools risk worsening inequities and excluding populations without access to devices or connectivity. To address this challenge, governments, international organizations, the private sector, nongovernmental organizations and partners must collaborate to build digital public infrastructure and diplomatic relations, and promote digital literacy. Digital health solutions should prioritize inclusivity, ensuring accessibility for all technological skill levels. Public–private partnerships and donor support can help bridge gaps and ensure no geographical area is left behind in the digital health revolution.

## Recommendations 

To advance digital health diplomacy, governments must collaborate to create multilateral frameworks addressing cross-border data sharing, privacy and technological standards. Strengthening digital public infrastructure in low- and middle-income countries should also be a priority for international organizations, ensuring underserved areas gain access to improved digital health services. Additionally, capacity-building programmes are essential, providing technical training for health professionals and diplomats on digital health tools. Public–private partnerships can help co-develop scalable digital health solutions, while monitoring and evaluation systems are needed to track the impact and inclusivity of these initiatives, ensuring they effectively reduce health disparities.

Achieving universal health coverage in the digital era requires innovation, collaboration, diplomatic relations and a strong focus on equity. The Alma-Ata Declaration, reinforced by the Astana Declaration in 2018, along with digital advancements, renews the commitment to health for all. Bridging the digital divide is both a technical and diplomatic challenge that requires global action, as digital health diplomacy fosters international cooperation to ensure these tools reach those most in need. Therefore, digital health diplomacy must remain central to international policy-making, offering a pathway to a sustainable future rooted in equity and community engagement.^13^

## References

[1] United Nations Secretary-General’s roadmap for digital cooperation. New York: United Nations; 2020. Available from: https://www.un.org/en/content/digital-cooperation-roadmap/ [cited 2024 Nov 12].

[2] Bjola C, Manor I. The rise of hybrid diplomacy: from digital adaptation to digital adoption. Int Aff. 2022 Mar 7;98(2):471–91. 10.1093/ia/iiac005

[3] Global strategy on digital health 2020–2025. Geneva: World Health Organization; 2021. Available from: https://www.who.int/publications/i/item/9789240020924 [cited 2024 Feb 10].

[4] Impact of digital technology development on diplomacy. New Delhi: International Journal of Scientific and Research Publications; 2023. Available from: https://www.ijsrp.org/research-paper-0523.php?rp=P13712858 [cited 2024 Feb 10].

[5] Barbosa da Silva J, Espinal M, Garcia-Saiso S, Fitzgerald J, Marti M, Bascolo E, et al. A digital transformation for primary health care. Bull World Health Organ. 2024 Jan 1;102(1):2–2A. 10.2471/BLT.23.290726 38164335 PMC10753283

[6] Kurbalija J. 12 AI and digital predictions for 2024. Malta: DiploFoundation; 2024. Available from: https://www.diplomacy.edu/blog/12-ai-and-digital-predictions-for-2024/ [cited 2024 Feb 13].

[7] Hassan K, Batool S. Evolving trends of digital diplomacy and its role in the achievement of foreign policy goals. J Policy Res. 2024;10(3):1–9. 10.61506/02.00311

[8] Hedling E, Bremberg N. Practice approaches to the digital transformations of diplomacy: toward a new research agenda. Int Stud Rev. 2021 Jun 24;23(4):1595–618. 10.1093/isr/viab027

[9] Manor I. The digital diplomacy bibliography [blog]. Exploring Digital Diplomacy; 2019. Available from: https://digdipblog.com/2019/04/04/the-digital-diplomacy-bibliography/ [cited 2024 Feb 11].

[10] Sustainable health agenda for the Americas 2018-2030. Washington, DC: Pan American Health Organization; 2017. Available from: https://www.paho.org/en/sustainable-health-agenda-americas-2018-2030 [cited 2024 Feb 11].

[11] Eight guiding principles of digital transformation of the health sector. A call to Pan American action. Washington, DC: Pan American Health Organization; 2021. Available from: https://iris.paho.org/handle/10665.2/54256 [cited 2024 Feb 11]

[12] CD59/6 - Roadmap for the digital transformation of the health sector in the Region of the Americas. Washington, DC: Pan American Health Organization; 2021. Available from: https://www.paho.org/en/documents/cd596-roadmap-digital-transformation-health-sector-region-americas [cited 2024 Feb 11].

[13] WHO called to return to the Declaration of Alma-Ata. International conference on primary health care. Geneva: World Health Organization; 2024. Available from: https://www.who.int/teams/social-determinants-of-health/declaration-of-alma-ata [cited 2024 Feb 12].

